# The Influence of Mixed Powder Ageing on the Structural, Chemical, and Crystalline Morphological Properties of the PA12 Used to Manufacture Laser Sintering

**DOI:** 10.3390/polym17050577

**Published:** 2025-02-22

**Authors:** Alejandro García Rodríguez, Edgar Espejo Mora, Marco Antonio Velasco Peña, Carlos Alberto Narváez Tovar

**Affiliations:** 1 Facultad de Ingeniería Mecánica, Universidad Santo Tómas, Carrera 9 No 51-11, Bogotá 110231, Colombia; 2 Mechanical and Mechatronics Engineering Department, Universidad Nacional de Colombia, Carrera 45 No 26-85, Building 407, Bogotá 111321, Colombia; eespejom@unal.edu.co (E.E.M.); canarvaezt@unal.edu.co (C.A.N.T.); 3 Technological Faculty, Universidad Distrital Francisco José de Caldas, Calle 13 No 31-75, Bogotá 110231, Colombia; mavelascop@udistrital.edu.co

**Keywords:** LS, PA12, used powder, particle size, DSC, XRD

## Abstract

This study investigated the effects of multiple laser sintering (LS) cycles on a polyamide 12 powder mixture of 70% recycled material and 30% virgin polymer. This research aimed to understand how repeated LS processing influences this specific powder mixture’s thermal and structural properties, which is crucial for optimising its performance in additive manufacturing applications. A thermal analysis revealed significant changes in the thermal behaviour of the powder over successive build cycles. Specifically, there was an observed increase in both the melting temperature and the crystallinity of the powder, alongside a notable decrease in the crystallisation temperature. These alterations suggest that the repeated LS cycles affect the thermal profiles and potentially enhance the material’s stability and usability in additive manufacturing processes. Additionally, a particle size distribution analysis indicated statistically significant differences between the initial and post-sintering states of the powder. These differences are significant as they can influence factors such as flowability and packing density, which are critical for the efficiency of additive manufacturing applications. Microscopic observations further revealed a strong correlation between the crystal morphologies and particle shapes, indicating that the structural changes occurring during processing are inter-related. The relationship underscores the importance of understanding microstructural evolution and the mechanical properties of the final printed products. These findings provide crucial insights into the microstructural evolution and thermal behaviour of recycled PA12 powder during multiple LS processing cycles. This study aids in developing practical strategies for sustainable and efficient powder recycling within the realm of additive manufacturing. By examining the intricate dynamics at play, the research opens avenues for enhancing the performance and environmental sustainability of 3D printing technologies, making them more accessible for various industrial applications.

## 1. Introduction

Laser sintering (LS) is the subject of extensive research, as the properties of materials, machinery, and postprocessing techniques are still being studied [1,2,3,4,5,6]. Research often focuses on studying the effects of the process on the powder, as this powder can be recycled and may reduce its environmental impact. This has drawn attention within the scientific community because of its potential for being environmentally friendly and sustainable [7,8,9,10]. Studies have investigated how process parameters impact the physicochemical properties of PA12 powder before and after sintering [11,12,13,14,15,16].

Damanhuri et al. [17] reported that they investigated the health effects of recycled powder on operators of laser sintering machines. The authors discovered that recycled powder contains a lower concentration of airborne particles than virgin powder, likely attributable to differences in particle size. Further research into the impact of particle size during mixing processes is necessary to ascertain potential health implications for equipment personnel.

Yang et al. [18] reported that the powder ageing time affects the thermal properties, particle size, and mechanical strength post-manufacturing. The authors observed that during recycling, there was an increase in fragmented particles, with their morphology transforming from round to irregular shapes across the build cycle. Additionally, the absence of humidity control between the build cycles reduced crystalline phases due to water exposure. Furthermore, carbonyl groups and hydrolysis were noted, possibly linked to a decline in mechanical properties. Notably, among the mixtures analysed, the 70:30 mixture demonstrated the best performance regarding mechanical properties following sintering.

Hejmady et al. [5] investigated the impact of laser parameters on particle size, crystallisation, and thermal conditions. This study indicates that non-isothermal crystallisation occurs, suggesting that traditional solidification models fail to account for the relationships between temperature, flow, and crystallisation kinematics. Additionally, we identified phase changes, with the α-phase predominating in sintered regions and the γ-phase present in unsintered areas of the powder. Furthermore, the viscoelastic properties of the polymer are influenced by the thermal effects resulting from powder recycling. The authors emphasise that imaging techniques are essential for a deeper understanding of the crystalline phenomena.

Weinmann et al. [19] highlight that incorporating new powder into the mixture can enhance the mechanical properties of the components. The powder utilised exhibits thermal effects that increase crystallinity while reducing the powder flow during the sintering process. The authors also suggest introducing chemical agents that can improve the viscous flow by breaking down polymer chains, resulting in a more amorphous material. This approach lowers costs and facilitates using more recycled materials, making the technique more environmentally sustainable.

Yang et al. [20] noted that varying the powder mix proportions between new and aged (10:90, 20:80, 30:70, 40:60, 50:50, 60:40, 70:30) influences a part of the performance. The repeated reuse of the powder leads to modifications at both the thermal and chemical levels, ultimately decreasing the mechanical strength of the components. The author notes that cyclic thermal stress degrades the polymer chains’ functional groups and bonds. Additionally, chemical degradation primarily involves the oxidation of key groups, which alters the molecular structure of the powder. Despite this observation, it was determined that specimens fabricated using 70:30 mixtures exhibited superior mechanical strength values compared to standard industrial samples. This finding suggests that powder degradation does not universally detriment the material properties, indicating the potential for optimised formulations in material engineering applications.

Martynková et al. [21] investigated using used powder at a 1:1 ratio, recovered and in a near-waste state. The findings suggest that secondary crystallisation may occur in powders utilised for pre-sintering, which hampers the sintering capacity of the material. This behaviour is because the laser needs more energy to achieve stable melt necks, forming internal and external pores in the final parts. Additionally, it was noted that with repeated build cycles, the powder exhibits reduced fluidity as smaller particles fragment and subsequently agglomerate with larger ones, which affects the uniformity of the sintering layers. In terms of morphology, the authors observed that used powder tends to show a decrease in particle roundness, resulting in irregular particle shapes. Despite these findings, the authors conclude that the effects of multiple build cycles remain to be fully assessed, particularly in determining the optimal cycle, after which the powder should no longer be reused.

The findings of these investigations indicate that even today, the powder’s chemical, thermal, and structural effects during the recycling process of PA 12 in laser sintering remain ambiguous in numerous respects. The authors hold differing views on the crystallisation of powders and the potential influence of thermal effects on particle size and shape. Additionally, it has been noted that much of the existing research tends to concentrate on concentrations exceeding 50% in virgin powder, likely because higher concentrations are often associated with the orange peel surface condition [22].

On the other hand, it has been shown in other research that parts manufactured with 70% used powder show good resistance and surface quality, reducing the need for new materials [23]. This combination of powders has not been studied to evaluate how its particle size, morphology, thermal, chemical, and structural properties change and whether this changes significantly in processes where more than two build cycles must be performed [24]. For this reason, this study examined the effects of using a 70% recycled and 30% virgin powder mixture before and after two build cycles on the structure, morphology, particle size, crystalline structure, and powder mixture composition, which could influence the printed parts.

## 2. Materials and Methods

### 2.1. Powder

Virgin and recycled EOS powder, PA 12-2200, was employed before and after sintering. The particle size was determined via SEM on a Vega 3 Tetscam instrument (Brno, Czech Republic) with a gold metallised sample. The particle size was evaluated via ImageJ software (version 1.54k 15 September 2024) with four SEM images. The mixture conditions (70% recycled before one sintering cycle, 30% virgin) were analysed before the sintering process. A sintering process was performed in an EOS Formiga P110 Velocid with standard machine parameters with a CO_2_ laser power of 30 W, a scan speed of 5 m s^−1^, a layer thickness of 0.12 powder mixture mm, and a build rate of 1.2 L/h. The powder population consisted of a mixture of powders located near the sintering regions of the part and those situated further from the laser. Once the samples were collected, they were combined in a powder mixer. The powder population followed the standard recycling procedure, mixing the unsintered powder close to the parts and far away. Ten grams of material was extracted, and the remaining was placed in the machine cube again. This population was then used for the next sintering cycle, and a sample was extracted from a retired mixed volume.

### 2.2. Infrared Spectroscopy (FTIR)

Infrared spectroscopy (FT-IR) was performed using a SHIMADZU IRAffinity-1S (Kyoto, Japan) spectrophotometer and the attenuated total reflection (ATR) technique. The measurement conditions were a spectral resolution of 8 cm^−1^, 64 scans per spectrum, and a wavenumber range of between 400 and 200 cm^−1^.

### 2.3. Differential Scanning Calorimetry (DSC)

A Mettler Toledo Differential Scanning Calorimeter (Giessen, Germany) 1-500/227 was used for thermal characterisation. The temperature range was between 25 °C and 200 °C, with a heating and cooling rate of 10 °C/min in a N_2_ atmosphere.

### 2.4. X-Ray Diffraction (XRD)

The powder crystalline structure was determined via a Philips diffractometer (Freiberg, Germany) with a 30 kV voltage and a 20 mA current, working in the Bragg–Brentano configuration with K alpha signals from the copper anode (γ = 0.1542 nm), between 2Θ values of 5–50° and a step size of 0.02°.

### 2.5. Microtome

The crystals of the powder particles were observed under a Karll Zeiss (Göttingen, Germany) polarised microscope. A wave polariser with a quarter-wave retarder was used. A microtome with macro search HP35 high-profile blades was used for sample cutting.

### 2.6. Statistical Analysis

Three quantities for each powder condition were randomly selected. Owing to the non-normality of the distributions, randomisation was performed, nonparametric data were evaluated via the Kruskal–Wallis (2500 particles per condition) test, and pairwise comparisons were conducted via the Bonferroni adjustment test.

## 3. Results and Discussion

### 3.1. Differential Scanning Calorimetry (DSC)

**Figure 1:**
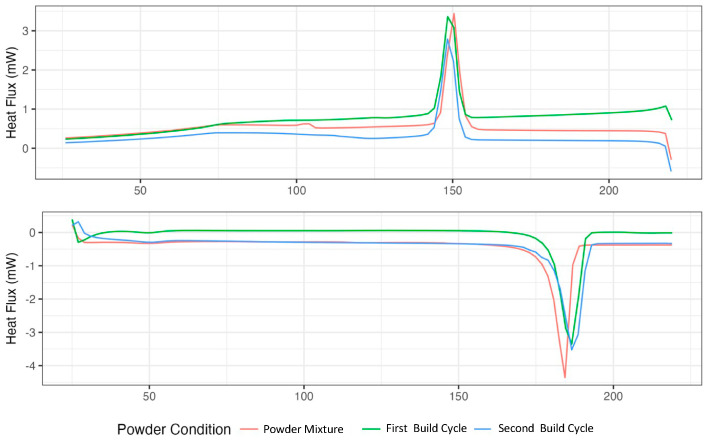
Exothermic and endothermic DSC spectra of each powder sample: powder mixture, after the first build cycle, and after the second build cycle.

The DSC spectrum of the PA12 virgin powder is displayed in red. In contrast, the powders after the first and second build cycle are displayed in blue and green, respectively (Figure 1). Figure 1 (upper) indicates endothermic behaviour, revealing that the crystallisation temperatures were lower in both the first and second sintering conditions than in the virgin conditions. Figure 1 (lower) shows the exothermic behaviour, and it is evident that the mixed used–virgin powder shifted toward lower melting temperatures than the used powder after the sintering cycles. Although the first and second sintering conditions had similar behaviours, the second condition slightly shifted toward higher melting temperature values.

Table 1 shows the powder’s crystallinity percentage (C.P.) values before and after the build cycle. Compared with the initial powder, the first and second sintering conditions resulted in 11.98% and 9.76% greater crystallinity percentages, respectively. The first and second build cycles increased the melting temperature (Tm) by 1.09 and 1.62%, respectively. Furthermore, the crystallisation temperature decreased by 0.66% in the first and 0.96% in the second samples compared with the mixed powder. Finally, the sintering window (S.W.) increased by 1.68% in the first and 2.27% in the second sintering.

An increase in the C.P. was observed after the sintering process, and this behaviour may be associated with the powder being subjected to temperatures above the Tc. Owing to these thermal conditions, the accumulation of polymeric chains in all the powder conditions was favoured, resulting in an annealing process [25,26]. Research, such as that of [18,27], shows a decay in the C.P. as a function of the number of build cycles. In contrast, the increase in the C.P. suggests that the powder’s state and proximity to the laser are essential. Short distances would lead to a more aggressive temperature change, decreasing the C.P. Therefore, the second process would have a lower C.P. value since it could have been in a zone closer to the laser and produced faster cooling.

**Table 1:** Crystallisation temperature (Tc), melting temperature (Tm), crystallinity percentage (C.P.), sintering window (S.W.), and sintering window for each powder condition: the powder mixture after the first build cycle and after the second build cycle. The thermal properties of each powder condition were compared with the reference values.

Property	Powder Condición	Present Study	Dadbakhsh et al. [27]	Cai et al. [28]	Yang et al. [18]	Yang et al. [29]	Yang et al. [26]
Tc (°C)	Powder mixture	150.28	158.35	148.20	148.23	141.94	155.46
	First build cycle	149.28	158.50	---	146.48	142.46	155.63
	Second build cycle	148.84	156.75		147.02	142.78	155.67
Tf (°C)	Powder mixture	182.58	184.05	188.90	186.38	182.53	180.15
	First build cycle	185.49	185.15	----	188.18	182.53	180.13
	Second build cycle	188.1	185.60		187.71	182.87	180.87
C.P (%)	Powder mixture	59.93	55.2 ± 0.5	48.20	46.60	45.48	51.98
	First build cycle	67.11	51.7 ± 0.1	---	50.02	44.92	51.17
	Second build cycle	65.78	51.1 ± 1.8		49.35	40.17	47.26
S.W (°C)	Powder mixture	18.44	17.80	---	25.70	29.92	---
	First build cycle	18.75	17.30		27.70	30.31	---
	Second build cycle	8.86	9.20		28.50	31.00	---

### 3.2. X-Ray Diffraction (XRD)

**Figure 2:**
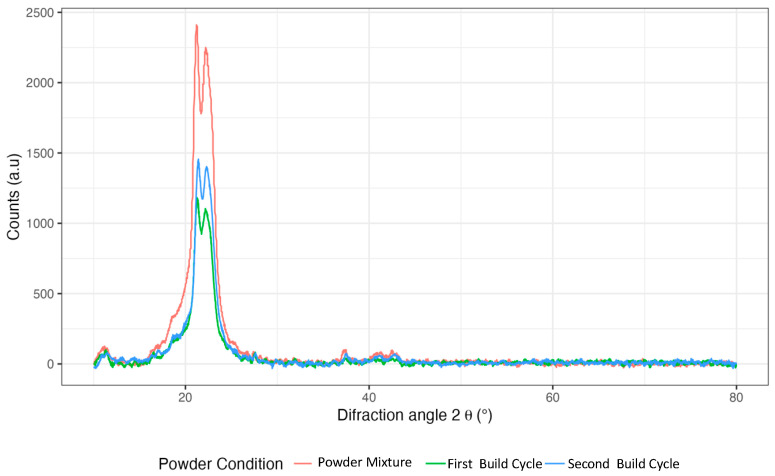
The DRX spectra of each powder sample: the powder mixture after the first and second build cycles.

Figure 2 shows the XRD spectrum of the PA12 powder samples. Two main signals corresponding to the phase were observed. The polyamide structure crystallises in the alpha phase (monoclinic) and gamma crystals (pseudohexagonal).

Table 2 below shows the angles corresponding to the phases of PA12. XRD revealed the characteristic gamma and alpha signals of PA12 [30]. There were no appreciable changes in either technique under the powder conditions, suggesting that the increase in crystallinity could be associated with agglomerated chains and is not due to the appearance of new phases in the powder material. A comparison of the X-ray diffraction (XRD) results with the differential scanning calorimetry (DSC) data reveals that, while no shifts in the diffraction angle were detected, the percentage of crystallinity in the powder was significantly influenced. These correlated observations suggest that, despite the absence of substantial phase transitions in the material, the degree of crystallisation in the powder may pose challenges to its flow characteristics during sintering. This outcome is particularly relevant, as a higher level of crystallinity typically necessitates an increased amount of energy for the powder’s partial melting.

### 3.3. Fourier Transform Infrared Spectroscopy (FTIR)

**Figure 3:**
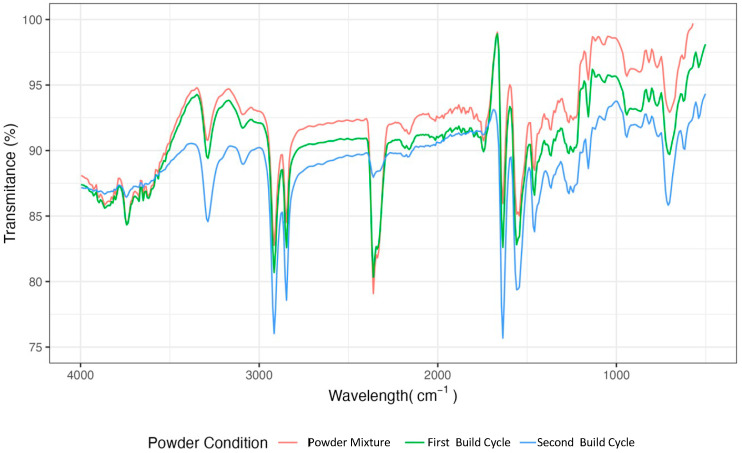
The FTIR spectra of each powder sample: the powder mixture after the first and second build cycles.

Figure 3 shows the IR spectra of PA powder under different conditions. Signals corresponding to the amide groups were observed. The amide IV group (bending) was observed at 632 cm^−1^; the amide III group (stretching) at 1275 cm^−1^ and 1578 cm^−1^; and the amide II group (bending) and the amide I group (stretching) at 1654 cm^−1^. No new signals were detected in the range of 1650 cm^−1^, which corresponds to the oxidation of the amide groups. Despite being near this range, amide group I did not exhibit any displacements or increases in the peak width as the build cycle progressed, suggesting that any potential degradation is insignificant in the IR spectrum.

Nevertheless, it was noted that the powder demonstrated moisture absorption during each of the build cycles, as indicated by the presence of an -OH band near 2500 cm^−1^ and -N_2_H and -COOH groups within the range of 3330–3500 cm^−1^, which are a result of water incorporation into the polyamide molecule [18]. In contrast, no crystalline or concurrent spectra changes were identified when these observations were compared with the results from the XRD and DSC analyses. The latter implies that, although the powder absorbed moisture, the quantity was insufficient to disrupt the chains and reduce the degree of crystallisation.

### 3.4. Size of the Particles

**Figure 4:**
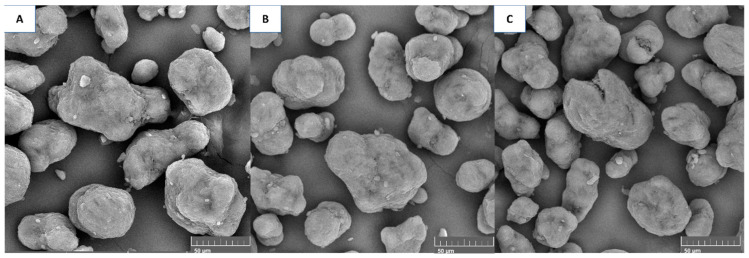
SEM images of the powder samples: (**A**) the powder mixture, (**B**) after the first build cycle, and (**C**) after the second build cycle.

Figure 4 shows the particulates for each of the powder conditions. The more build cycles the powder underwent, the more irregularly shaped the particles tended to become.

**Figure 5:**
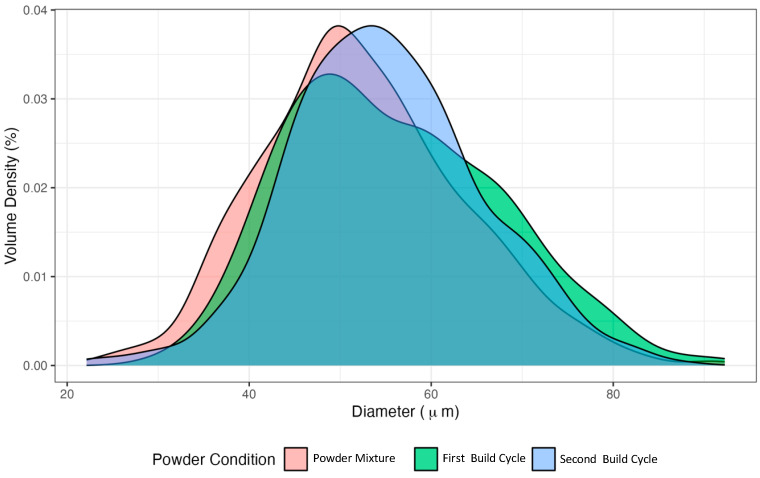
Diameter distribution spectrum of each powder condition: the powder mixture before and after the first and second build cycles.

Figure 5 shows the particle diameter distribution for each of the PA12 powder conditions. The powder diameter size distribution had the highest particle density, corresponding to values close to 50 µm. In analysing the particle size diameter distribution, it is evident that before the sintering process, a distinct peak is observed within the range of 30–40 μm. Conversely, the particle diameter distributions established post-sintering demonstrate peaks within the range of 60 μm to 70 μm. This phenomenon suggests that a substantial quantity of particles exist at diameters more petite than the average before the build cycle. Following the sintering processes, these smaller particles are likely to coalesce, partially or entirely, with those of average diameters, thereby accounting for the observed shifts towards larger diameter values.

According to Table 3, the diameter of the particle increases by approximately 6.66% after one sintering cycle and 4.99% after two build cycles compared with that of the powder before the build cycle (Table 1). A Kruskal–Wallis nonparametric statistical test revealed statistically significant differences in the particle size distribution (*p* = 5.91 × 10^−7^). The Bonferroni post hoc comparison test showed a significant difference in the particle diameter between the initial powder and the first sintering powder (*p* = 3.9 × 10^−6^) and between the initial powder and the second sintering powder (*p* = 1.3 × 10^−4^).

### 3.5. Morphology of the Particle Crystal

**Figure 6:**
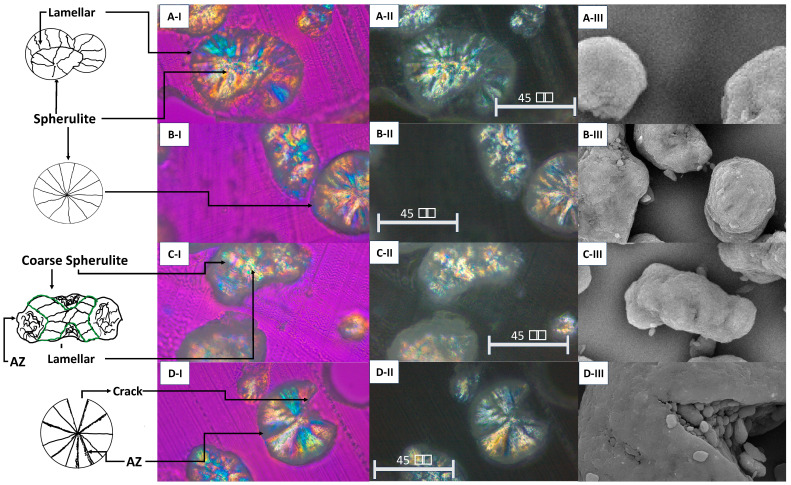
The morphologies of the powder particles and their crystals. The (I) images were taken with polarised light with a quarter-wave retarder. The (II) images were taken only with polarised light, and the (III) images were SEM images. (**A**) Potato-type morphology, (**B**) circular-type morphology, (**C**) irregular-type morphology, (**D**) circular-particle-type crack.

Figure 6 shows crystal morphology as a function of the particle morphology. Figure 6A shows a particle potato morphology with spherulite crystals in the centre, which are of a greater diameter and show preferential lamellar growth in the particle limits. Figure 6B shows a spherical particle with assertive spherulitic behaviour. The black zones at the crystal boundaries could be the particle amorphous zones (AZs). Figure 6C shows an irregular particle that presents a coarse spherulitic crystal. In addition, lamellar-type crystals were observed inside the spherulite particles. Finally, in Figure 6D, a circular particle with a crack was observed. The fracture was intercrystalline, and spherulitic-lamellar crystals were present in the particle.

The particle size of the powder significantly changed, as shown by comparing the powder mixture with the powder conditions used. This change was not just in size; the particle morphology also varied. Initially, circular particles were found in the virgin powder, but more irregular and fractured shapes appeared with recycling. These changes impacted sample defects such as fusion issues, porosity, and an orange peel texture [27,32].

This morphological change was associated with the crystal shape. The spherulitic crystals were generally symmetrical. In contrast, the potato morphology had a spherulite section where lamellar structures, resulting from the thermal process, followed. Finally, the irregular particles subjected to considerable thermal changes exhibited a thicker spherulitic behaviour with lamellar subcrystals.

On the other hand, particle fracture is a phenomenon that is not clear today. Some theories associate this cracking with the thermal effect [33] and mechanical damage [34]. This research proposes a combination of both approaches. Although the fractured particle crystals are mainly spherulites, lamellar sections are also present. These thermal changes during sintering create crystalline gradients, increasing the resistance of some zones. When the blade sweeps the powder, stress on weaker areas can cause intercrystalline fractures at the crystal boundaries, which are considered amorphous.

**Figure 7:**
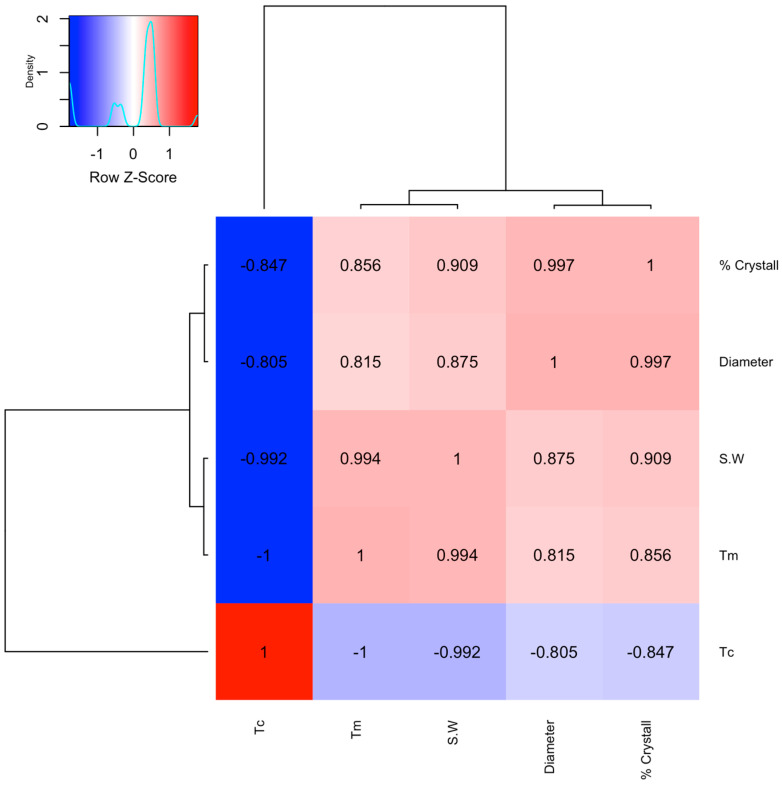
Correlation between the thermic properties and the diameter of the particle.

This study revealed strong statistical correlations between the thermal variables, particle size, and morphology. Figure 7 shows that the properties present a proportional correlation, except for Tm-Tc, which offers an inverse relationship. This behaviour should be studied as a function of the proximity of the laser in the sintering chamber to determine whether it is replicable or if the used powder has significant anisotropic conditions.

## 4. Conclusions

Before and after one and two build cycles, the PA12 2200 powder was thoroughly analysed in its mixed mixture. The chemical composition remained unchanged, but the particle size and morphology significantly changed. A strong link between powder morphology and thermal properties led to a hypothesis explaining the particles’ cracking. This study highlighted how sintering affects the particle size and structure, which are key factors in sample defects. Future research will explore the relationship between the crystal shape and the laser distance to understand various behaviours in raw material studies.

## Figures and Tables

**Table 2:** The comparison of the thermal properties of each powder condition with the literature values.

Phase PA 12	Powder Condition	Present Study	Yao et al. [31]	Cai et al. [28]	Yang et al. [29]
Gamma (100)	Powder mixture	21.221	20.3	21.2	20.3
	First build cycle	21.283			
	Second build cycle	21.393			
Alpha (010/110)	Powder mixture	22.253	23.2	22.1	23.9
	First build cycle	22.274			
	Second build cycle	22.340			

**Table 3:** The particle diameter of the powder before and during the first and second sintering, with bibliography values.

Condition	Present Study (μm)	Cai et al. [28]	Dadbakhsh et al. [27]	Yang et al. [29]	Jiang et al. [11]	Alo et al. [32]
Powder Mixture	52.5	52.6	55–60	50–60	63	55.75
First Build Cycle	56.0					
Second Build Cycle	55.1					

## Data Availability

The original contributions presented in this study are included in the article. Further inquiries can be directed to the corresponding author.

## References

[1] Rodríguez A.G., Mora E.E., Velasco M.A., Tovar C.A.N. (2023). Mechanical properties of polyamide 12 manufactured by means of SLS: Influence of wall thickness and build direction. Mater. Res. Express.

[2] Rodriguez A.G., Peña M.A.V., Narváez-Tovar C.A., Mora E.E. (2024). Experimental analysis of failure behaviour of PA12 specimens manufactured by SLS as a function of wall thickness and build direction. Rapid Prototyp. J..

[3] Traxel K.D., Malihi D., Starkey K., Bandyopadhyay A. (2020). Model-driven directed-energy-deposition process workflow incorporating powder flowrate as key parameter. Manuf. Lett..

[4] Baserinia R., Brockbank K., Dattani R. (2022). Correlating polyamide powder flowability to mechanical properties of parts fabricated by additive manufacturing. Powder Technol..

[5] Hejmady P., van Breemen L.C.A., Hermida-Merino D., Anderson P.D., Cardinaels R. (2022). Laser sintering of PA12 particles studied by in-situ optical, thermal and X-ray characterization. Addit. Manuf..

[6] Kruth P., Wang X., Laoui T. (2003). Lasers and materials in selective laser sintering. Assem. Autom..

[7] Greiner S., Jaksch A., Cholewa S., Drummer D. (2021). Advanced Industrial and Engineering Polymer Research Development of material-adapted processing strategies for laser sintering of polyamide 12. Adv. Ind. Eng. Polym. Res..

[8] Sivadas B.O., Ashcroft I., Khobystov A.N., Goodridge R.D. (2021). Laser sintering of polymer nanocomposites. Adv. Ind. Eng. Polym. Res..

[9] Yap C.Y., Chua C.K., Dong Z.L., Liu Z.H., Zhang D.Q., Loh L.E., Sing S.L. (2015). Review of selective laser melting: Materials and applications. Appl. Phys. Rev..

[10] Yusoff W., Pham D.T., Dotchev K. Effect of Employing Different Grades of Recycled Polyamide 12 on the Surface Texture of Laser Sintered (Ls) Parts. Proceedings of the International Conference on Advances in Materials & Processing Technology (AMPT 2009).

[11] Jiang X., Shen W., Jiang L., Qin H. (2022). Effects of Particle Size Distribution and Impact Speed on Printing Quality in Direct Energy Deposition. Manuf. Lett..

[12] Starr T.L., Gornet T.J., Usher J.S. (2011). The effect of process conditions on mechanical properties of laser-sintered nylon. Rapid Prototyp. J..

[13] Salmoria G.V., Leite J.L., Paggi R.A. (2009). The microstructural characterization of PA6/PA12 blend specimens fabricated by selective laser sintering. Polym. Test..

[14] Chou B.F.E.K. (2022). Build surface study of single-layer raster scanning in selective laser melting: Surface roughness prediction using deep learning. Manuf. Lett..

[15] Gawade V., Galkin G., Guo Y.B., Guo W.G. (2022). Quantifying and modeling overheating using 3D pyrometry map in powder bed fusion. Manuf. Lett..

[16] Wegener M.S.E.K. (2016). Additive Manufacturing: Polymers applicable for laser sintering (LS). Procedia Eng..

[17] Damanhuri A.A.M., Hariri A., Ghani S.A., Mustafa M.S.S., Herawan S.G., Paiman N.A. (2021). The Effects of Virgin and Recycled PA12 Powders in SLS Processes on Occupational Exposures. Int. J. Environ. Sci. Dev..

[18] Yang F., Zobeiry N., Mamidala R., Chen X. (2022). A review of aging, degradation, and reusability of PA12 powders in selective laser sintering additive manufacturing. Mater. Today Commun..

[19] Bonten S.W.E.C. (2020). Recycling of pa12 powder for selective laser sintering. AIP Conference Proceedings.

[20] Yang F., Jiang T., Lalier G., Bartolone J., Chen X. (2020). A process control and interlayer heating approach to reuse polyamide 12 powders and create parts with improved mechanical properties in selective laser sintering. J. Manuf. Process..

[21] Martynková G.S., Slíva A., Kratošová G., Barabaszová K.Č., Študentová S., Klusák J., Brožová S., Dokoupil T., Holešová S. (2021). Polyamide 12 materials study of morpho-structural changes during laser sintering of 3d printing. Polymers.

[22] Launhardt M., Fischer C., Drummer D. (2015). Research on the Influence of Geometry and Positioning on Laser Sintered Parts. Appl. Mech. Mater..

[23] Pham D.T., Dotchev K.D., Yusoff W.A.Y. (2008). Deterioration of polyamide powder properties in the laser sintering process. Proc. Inst. Mech. Eng. C J. Mech. Eng. Sci..

[24] Schuk M.K. (2018). Laser Sintering with Plastics.

[25] Majewski C.E., Zarringhalam H., Hopkinson N. Effects of degree of particle melt and crystallinity in SLS Nylon-12 parts. Proceedings of the 19th Annual International Solid Freeform Fabrication Symposium.

[26] Yang W., Liu F., Zhang J., Zhang E., Qiu X., Ji X. (2017). Influence of thermal treatment on the structure and mechanical properties of one aromatic BPDA-PDA polyimide fibers. Eur. Polym. J..

[27] Dadbakhsh S., Verbelen L., Verkinderen O., Strobbe D., Van Puyvelde P., Kruth J.P. (2017). Effect of PA12 powder reuse on coalescence behaviour and microstructure of SLS parts. Eur. Polym. J..

[28] Cai C., Tey W.S., Chen J., Zhu W., Liu X., Liu T., Zhao L., Zhou K. (2020). Comparative study on 3D printing of polyamide 12 by selective laser sintering and multi jet fusion. J. Mech. Work. Technol..

[29] Yang F., Jiang T., Chen X. (2020). Process control of surface quality and part microstructure in selective laser sintering involving highly degraded polyamide 12 materials. Polym. Test..

[30] El Magri A., Bencaid S.E., Vanaei H.R., Vaudreuil S. (2022). Effects of Laser Power and Hatch Orientation on Final Properties of PA12 Parts Produced by Selective Laser Sintering. Polymers.

[31] Yao B., Li Z., Zhu F. (2020). Effect of powder recycling on anisotropic tensile properties of selective laser sintered PA2200 polyamide. Eur. Polym. J..

[32] Alo O.A., Otunniyi I.O., Mauchline D. (2022). Aging due to successive reuse of polyamide 12 powder during laser sintering: Extrinsic powder properties and quality of sintered parts. MATEC Web Conf..

[33] Verbelen L., Dadbakhsh S., Van Den Eynde M., Kruth J.P., Goderis B., Van Puyvelde P. (2016). Characterization of polyamide powders for determination of laser sintering processability. Eur. Polym. J..

[34] Balemans C., Jaensson N.O., Hulsen M.A., Anderson P.D. (2018). Temperature-dependent sintering of two viscous particles. Addit. Manuf..

